# Draft Genome Sequence of a Tetracycline-Resistant Plesiomonas shigelloides Strain Isolated from Aquaculture-Reared Tilapia

**DOI:** 10.1128/MRA.00832-18

**Published:** 2018-07-19

**Authors:** Ana F. M. Martins, Herrison Fontana, Beatriz M. Moreira, Raquel R. Bonelli

**Affiliations:** a Laboratório de Investigação em Microbiologia Médica, Instituto de Microbiologia Paulo de Góes, Universidade Federal do Rio de Janeiro, Rio de Janeiro, Brazil; Indiana University Bloomington

## Abstract

We hereby present the 3.7-Mb draft genome sequence of Plesiomonas shigelloides strain FM82, isolated from a tilapia (Oreochromis niloticus) reared in a fish farm in Rio de Janeiro, Brazil. P. shigelloides strain FM82 carries antimicrobial resistance, biofilm, and CRISPR-related genes.

## ANNOUNCEMENT

Plesiomonas shigelloides is a Gram-negative rod-shaped mesophilic bacterium with a facultative anaerobic, chemoorganotrophic energy metabolism (1). Although the taxonomic positioning of P. shigelloides has long been debated, the Plesiomonas genus is currently a member of the Enterobactereaceae family. The genus comprises a single species (2). The metabolic property of being oxidase positive establishes a difference from other members of the family, but the enterobacterial common antigen (ECA) is present (3).

This bacterium is frequently isolated from aquatic environments and is part of the intestinal microbiota of fish (4). Disease in humans has been reported, mainly associated with gastrointestinal illnesses, but extraintestinal infections also occur (5).

Limited, and occasionally conflicting, information is available regarding the pathogenic potential, virulence factors, and antimicrobial susceptibility of P. shigelloides (2, 6, 7). Enterotoxic and cytotoxic mechanisms have been described, as has its ability to adhere and invade enterocyte cells *in vitro* (2, 8). Except for some penicillins, P. shigelloides is generally susceptible to most available antimicrobials, such as tetracyclines and quinolones (9, 10). Nonetheless, antibiotic susceptibility described for strains is highly variable, and no robust correlation has been established between susceptibility profiles and strain origin or serotype (2).

P. shigelloides strain FM82 was isolated in an agar medium supplemented with tetracycline (4 µg/ml). The strain was obtained from a bowel sample of a tilapia (Oreochromis niloticus) destined for human consumption that was reared in a natural earthen pond in a high-density floating cage in Rio de Janeiro, Brazil. This project (process number 01200.001568/2013-87) was approved by the Ethics Committee for the Use of Animals of the Centre of Health Sciences at the Federal University of Rio de Janeiro and registered at the National Council for the Control of Animal Experimentation (reference number 085/14). Euthanasia of the tilapia was performed according to the University of Washington policy for the euthanasia of finfish species (http://depts.washington.edu/oawhome/wordpress/wp-content/uploads/2013/10/Euthanasia-of-Fish_Species-2013.pdf).

Genomic DNA was extracted using a DNeasy blood and tissue kit (Qiagen, Inc., Valencia, CA, USA). Whole-genome sequencing was performed with Illumina MiSeq 2500 platforms using 2 × 250-bp paired-end reads. Raw reads were quality checked with FastQC v.11.7 (https://www.bioinformatics.babraham.ac.uk/projects/fastqc/), and low-quality reads were trimmed using Trimmomatic v. 0.36 (11). Subsequently, the quality-filtered reads were *de novo* assembled using SPAdes v.3.11.1 (12), and contigs were functionally annotated with Rapid Annotation using Subsystem Technology (RAST) v. 2.0 (13). The resulting assembly yielded 193 contigs, totaling 3,729,830 bp, with a G+C content of 51.5%, and containing 3,205 coding sequences and 58 RNA genes.

ResFinder v. 3.0 (14) identified the tetracycline resistance genes *tet*(A) (GenBank accession number AJ517790) and *tet*(D) (GenBank accession number AF467077) and the fluoroquinolone resistance mutation S83I in *gyrA*. RAST annotation revealed genes coding for biofilm formation proteins PgaA, PgaB, PgaC, and PgaD, macrolide-specific efflux proteins MacA and MacB, and toxin-antitoxin system proteins HigA, YoeB, and YefM. CRISPRFinder (15) revealed genes coding for clustered regularly interspaced short palindromic repeat (CRISPR)-associated proteins Cas1, Cas6, and Csx3.

In summary, this draft genome sequence provides valuable information for a better understanding of antimicrobial resistance reservoirs in aquaculture settings and the zoonotic potential of P. shigelloides.

### Data availability.

The whole-genome shotgun project has been deposited at DDBJ/ENA/GenBank under the accession number PYSI00000000. The version described in this paper is version PYSI01000000. The data discussed here can be accessed at https://www.ncbi.nlm.nih.gov/genome/17449?genome_assembly_id=375113.
